# The Renal Form of Enteric Fever

**Published:** 1898-12

**Authors:** J. C. Wilson

**Affiliations:** Philadelphia


### The Renal Form of Enteric Fever.

BY J. C. WILSON, M. D., PHILADELPHIA.

The prominence of certain symptoms in cases of enteric fever, the evidences of intense implication of special organs, and occasional modifications in the course of the attack, have given rise to various attempts to arrange the cases in categories and to describe special forms of the disease.

We find in systematic writings upon the subject descriptions more or less complete of the following forms of enteric fever: 1. The mild, or typhus levissimus. 2. The latent or ambulatory form —walking typhoid. 3. The abortive form. 4. A febrile enteric fever. 5. The enteric fever of childhood—so-called infantile remittent fever. 6. Enteric fever in the aged. 7. Hemorrhagic typhoid, the last being a rare form, commonly fatal, characterized by hemorrhages into the skin and from mucuous surfaces, and corresponding to the hemorrhagic forms of variola, measles, and other infections. These categories are similar to the groupings of cases made by common consent in the description of other acute diseases, and unquestionably serve a useful purpose. When, however, we observe a disposition to set other lines of division and to group the cases upon a different system of classification, we are impressed with the unsatisfactory results of nosological refinements. To describe bilious and sudoral forms and to speak of ataxic and adynamic types, is neither scientific nor convenient; nor, for the student, does it simplify the subject to constitute distinct varieties, such as pleural, pulmonary, cerebro-spinal, and renal. Nevertheless, aberrant forms, of which these terms are descriptive, are encountered. They are too rare to constitute varieties of the disease; too common to be disregarded in systematic descriptions.

Some years ago a woman, aged about forty years, was admitted to my service in the hospital of the Jefferson Medical College, who presented the general symptoms of enteric fever with the physical signs of a croupous pneumonia. The illness began suddenly, with chill and very high temperature. The case terminated fatally. Upon autopsy the abdominal lesions of enteric fever were found, and Eberth's bacilli were recovered from the pulmonary exudate.

The wife of a physician was taken suddenly ill with intense headache, vomiting, and painful rigidity of the muscles of the back of the neck. The heart's action was feeble and irregular. There was active febrile movement of remittent type. At the end of a week the cerebro-spinal symptoms abruptly ceased, rose-spots appeared, the spleen bcame enlarged, and the case ran the course of an uncomplicated enteric fever.

A Russian boy, aged four years, was admitted to the Pennsylvania Hospital on the fourth day of an illness attended with pain in the limbs and swelling and tenderness of the belly. Upon admission there was slight painful rigidity of the muscles of the back of the neck, together with spasmodic contractions of the muscles of the arms and hands. Except dilatation, there was no pupillary symptoms, no strabismus, no trismus. There was no disease of the ears. A few hours after admission the patient's temperature fell from 102 degrees to 95 degrees F. It rapidly rose again. The child was fretful, and preferred to lie upon his side, with his limbs strongly flexed. He cried out with pain upon being moved. Ankle-clonus was present, and the knee-jerks were increased. A small amount of albumin, with hyaline and granular casts, appeared in the urine. There were one or two hepatic vesicles upon the lips, and a few pustules upon the occipital region and upon the trunk; the tongue was moist, thickly coated with a white, pasty fur; it was red at the tip and edges. Upon the third day after admission a group of typical rose-spots appeared upon the abdomen and chest, and the spleen was made out to be enlarged. About this time there was rapid amelioration of the nervous symptoms; the reflex phenomena subsided; pain on movement disappeared, and the case presented symptoms of a typical enteric fever of moderate severity in childhood. Treatment, systematic cold bathing. Convalescence was retarded by furunculosis.

The following case, recently seen in consultation with Dr. Byers and Dr. Hobson, is an example of the renal form of enteric fever—nephro-typhoid:

A lad, aged nineteen years, salesman, who had never had enteric fever and whose previous health had been excellent, began, about December 10, 1897, to complain of weakness and loss of appetite. A few days later he had headache and was feverish in the evening. It was noticed that he was unusually pale. December 24th he was obliged to abandon his work and remain in bed. About this time headache became severe.

From this time on there was continued fever, the temperature ranging from 99.3 degrees F. as a single minimum in the morning to 105 degrees F. as a maximum in the evening, until the fifty-fourth day, when it became normal and remained so. During the greater part of this period the temperature fluctuated between 102 degrees and 104 degrees F. From the time the patient was obliged to remain in bed until January 8th, namely, a period of fifteen days, the symptoms were those of an acute nephritis. The urine varied in specific gravity from 1010 to 1030, and showed large amounts of albumin, together with erythrocytes, leucocytes, epithelial cells, and granular casts. The breath was urinous, the skin hot and dry, and there were delirium and occasional vomiting, with a slight puffiness under the eyes, but upon the most careful examination no œdema elsewhere. An examination of the eye-grounds by Dr. Hansell showed engorgement of the venous circulation, but no retinitis nor any signs of past trouble. The amount of urine was about twenty-five fluidounces in twenty-four hours.

The patient did not improve under treatment directed to this condition, including calomel and saline purging, hot-air baths, and cut cups in the lumbar region, although under this treatment the quantity of urine voided was increased to sixty ounces in twenty-four hours. He became greatly emaciated and weak. On January 8th the following note was made: General condition much improved, but the patient continues to be extremely weak. The temperature now ranges between 99 degrees and 101 degrees F. The amount of urine voided amounts to eighty or ninety ounces in each twenty-four hours. The albumin is greatly diminished, casts have almost entirely disappeared, one only now and then being found in the field. A few blood corpuscles are occassionally seen. For the first time the patient on this date passed blood in the stools.

Small amounts of blood continued to be present in the discharges from the bowels for eight days. Prior to this time the case had been carefully studied from the standpoint of a possible enteric fever. Abdominal symptoms were not, however, present, rose-spots could not be found, nor enlargement of the spleen made out. An examination of the blood now, however, gave a positive reaction to the Widal test. A liquid diet consisting chiefly of milk had been administered from the beginning of the attack.

On January 26th it was noted that the urine was free from albumin, but still contained a few erythrocytes and leucocytes. The temperature had fallen to 99.3 degrees F. From this point the temperature began steadily to rise to the former level, the patient becoming delirious, albumin reappeared, together with granular casts, leucocytes, and erythrocytes. On February 2d there were diarrhœa and tympany, but no spots. On the 3d a rather copious crop of the rose-spots of enteric fever appeared. From this time on until February 19th the progress of the case was that of enteric fever. Fresh crops of spots appeared, increase in the area of splenic dulness could be made out, the tendency to diarrhœa persisted. Albumin, casts, and blood-corpuscles disappeared from the urine. Finally the patient developed the intense hunger so characteristic of the early convalescence. At the time of writing, April 15th, the urine continued to be normal.

In this case the extended febrile movement covers an obscure primary attack in which the kidneys manifestly bore the brunt of the infection, and a relapse occurring without interval—so-called intercurrent relapse—in which the ordinary symptom-complex of enteric fever is present with renewed evidences of inflammation of the kidney. Such cases are extremly rare. Their recognition is, however, of great importance.

Aside from the general theoretical importance of a correct diagnosis, we must consider here certain practical points. First, while the kidneys may, as in the foregoing instance, for a time bear the brunt of the attack, the ordinary intestinal lesions may also be present, and in this particular case the occurrence of hemorrhage was, without doubt, the evidence of deep ulceration attended with the danger of perforation. There are, then, practically two clinical dangers in these cases which render the recognition of their essential nature necessary to the welfare of the patient: first, the danger of the administration of an improper diet; and, second, that of the improper use of purgatives. The mistake of regarding a case of enteric fever of the renal form as one of acute nephritis occasions but little danger on the part of the prudent practitioner, since the regulation of the nourishment and a diet consisting largely of milk or dilute porridge would enter into the scheme of management; but there are those who are not altogether strict in regard to the diet of their cases of acute nephritis, by whom fruits, certain vegetables, or other solid food might be permitted.

The danger from an error in diagnosis is altogether great with reference to the use of purgatives, since the administration of calomel in full doses, and salines, enters very largely into accepted plans of treatment for acute nephritis. The diagnosis is, however, as difficult as it is important, and must remain in many cases an impossible one until the disease has made some progress.

Of even greater importance is the danger of communication of the disease which attends an error in diagnosis. From the standpoint of preventive medicine the failure to recognize a case of enteric fever is attended with risks to the community enormously greater than any danger that may affect the interests of the individual patient. The final destruction of the infecting principle in the stools and urine by efficient disinfection means the arrest of the spread of the disease upon the spot so far as any particular case goes. To neglect this procedure amounts practically to a crime against the State. The differential diagnosis between enteric fever and any other disease with which it may be confounded involves from this point of view enormous responsibility.

The following case will illustrate the difficulty of diagnosis:

I saw recently in consultation with a medical friend, at about the end of the second week of her illness, an unmarried woman, aged thirty-six years, who had been taken sick gradually with headache, great weakness, tendency to diarrhœa, and other evidences of enteric fever. The spleen was enlarged. There were no rose-spots. The tongue was red at the edges and tip and heavily coated with a yellowish-white fur. In the course of a routine examination the evidences of an acute nephritis had been discovered in the urine. There was slight prætibial œdema. The temperature ranged between 102 and 104 degrees F. The general appearance of the case was that of enteric fever of mild type. At my suggestion, the agglutinating power of the blood-serum upon Eberth's bacilli was tested (Widal test). About seven weeks later I was informed by the physician in charge that the convalescence of the patient was practically assured. In addition he wrote as follows: "After you saw the case her temperature pursued a zigzag, gradually declining and becoming normal about the twenty-second day after the nurse arrived," about the twenty-ninth day of the attack. "There was some mild delirium, and after some days the tongue became dry and glazed. The albumin disappeared as the fever subsided, but after some days there developed a phlebitis of the left leg, after which albumin was again temporarily present in the urine. No rose-spots were discovered. The Widal test was negative. While willing to admit that the Scotch verdict of 'not proven' is applicable to this case, I still feel that it was one of typhoid infection."

In this case a repetition of the Widal test at a later period was unfortunately not made. The patient ultimately entirely recovered, with permanent disappearance of albumin.

The agglutination-test is of the utmost importance in finally settling the question of diagnosis in doubtful cases. In the form of the disease under consideration, however, its value was impaired by the fact that in a certain proportion of the cases the power of arresting the motility of the typhoid bacilli and causing agglutination does not show itself until the disease has made considerable progress —not earlier in some cases than the end of the second or some period in the course of the third week.

It is of practical importance, then, to treat all doubtful cases in which for the time being the differential diagnosis between subacute or acute nephritis and enteric fever cannot be made as possible cases of enteric fever. Certainly this method of management is imperative as regards the regulation of the diet and the disinfection of the stools and the urine—matters which involve no risk in any case and which recommend themselves for acceptance in doubtful cases in view of the fact that in the majority of instances in which this particular differential diagnosis is in question the sickness will ultimately prove to be enteric fever.—*Amer. Jour. Med. Science.*

